# QLiS – development of a schizophrenia-specific quality-of-life scale

**DOI:** 10.1186/1477-7525-10-61

**Published:** 2012-06-07

**Authors:** Michael Franz, Michael Fritz, Bernd Gallhofer, Thorsten Meyer

**Affiliations:** 1 Centre for Psychiatry, Justus-Liebig University, Giessen, Germany; 2 Vitos Klinikum Kurhessen, Bad Emstal, Germany; 3 Vitos Forensic Psychiatric Hospital, Haina, Germany; 4 Institute for Epidemiology, Social Medicine and Health Systems Research, Hannover Medical School, Hannover, Germany

**Keywords:** Quality of life, Personal satisfaction, Schizophrenia, Questionnaires, Outcome assessment

## Abstract

**Background:**

The aim of the project was to develop an instrument for the assessment of subjective quality of life specific to schizophrenic persons on the basis of patients’ views on their own life and on sound psychometric principles.

**Methods:**

The project applied a six-step multiphase development process with six distinct studies. (1) The elicitation of schizophrenic persons’ views on their quality of life was based on open-ended interviews with interviewees from different settings (acute ward inpatients, long-term care patients, community care patients; n = 268). (2) A cross-sectional study with schizophrenic and healthy persons was conducted to quantify the relative importance of the various aspect of quality of life that emerged from the qualitative study (n = 143). (3) We conducted an empirical comparison of response formats with schizophrenic persons (n = 32). (4) A scale construction- and reliability-testing study was performed (n = 203) as well as (5) a test-retest reliability study (n = 49). (6) The final questionnaire (QLiS, quality of life in schizophrenia) was tested in an additional study on convergent and discriminant validity (n = 135).

**Results:**

The QLiS comprises 52 items (plus 2 optional items related to work) in 12 subscales: social contacts, appreciation by others, relationship to family, appraisal of pharmacotherapy, appraisal of psychopathological symptoms, cognitive functioning, abilities to manage daily living, appraisal of accommodation/housing, financial situation, leading a ’normal‘ life, confidence, general life-satisfaction. An item response format with four response categories was preferred by the schizophrenic persons. The mean values of the subscales clustered around the theoretical mean of the subscales and only minimal ceiling effects were found. The reliability (test-retest-reliability and internal consistency) was with one exception > .70 for all subscales.

**Conclusion:**

Taking the low numbers of items per subscale into account, the QLiS can be regarded as an accurate assessment instrument of subjective quality of life in schizophrenia with good content validity.

## Background

Research on quality of life (QoL) in psychiatry is still growing in the first decade of this century. This shows that it has not been a mere fashion but has found its place, especially in evaluative and outcomes research (e.g. [1,2]), and, more recently, in health economics or utility-based approaches (e.g. [3,4]). However, QoL is – still – an equivocal term with substantially different meanings, characterizing a “field of interest” [5] and not a single scientific construct. Since it is not applied consistently with regard to its meaning, it has to be defined before use. Repeatedly it has been argued that QoL research needs a better theoretical foundation, both within psychiatric research as well as in other medical fields [6-10].

Generally speaking, in doing QoL research, we have to decide on at least two key questions: 1. What constitutes QoL for a given patient group? This question refers to the important components of QoL in these patients. 2. Who judges whether a patient’s quality of life is good or bad? Beginning with social-indicator research, there has been a long tradition of using social standards – such as income level, number of friends, or personal assets – as a gauge of quality of life. However, a central hallmark of QoL in medical research has been the integration of the person’s individual perspective [11], meaning that the person him- or herself decides on the quality of his or her life. Satisfaction ratings are an expression of a widely used criterion to judge one’s own life, either by rating satisfaction in different life domains or by rating general life satisfaction. These satisfaction ratings are part of a number of different QoL instruments in psychiatric research, e.g. the Lehman Quality of Life Interview [12], which has been adapted from the instruments used in the social-indicator-research tradition in the general (US) population. Other adaptions of satisfaction ratings to psychiatry are the Lancashire Quality of Life Profile [13,14] and the Manchester Short Assessment of Quality of Life [15]. We will refer to this approach, in which the patients themselves appraise their QoL, as subjective quality of life (SQoL). This should not be confused with self-reported QoL, or to use a more-recently established term, patient-reported outcome (PRO) [16], which refers to the source of information. In recent years, generic health-related QoL instruments, such as the WHOQOL [17], the Short-Form 36 [18], representing PRO measures, and also the EQ-5D [19](a health economic utility measure) became popular in the assessment of aspects of QoL in psychiatric research. In these measures, aspects of SQoL are measured only indirectly, since they are mainly based on reports about aspects of QoL especially related to functioning and not on appraisal of different aspects of life.

Despite the presumption that SQoL instruments should be based on the perceptions of the persons themselves [11], quite surprisingly the views of schizophrenic patients on their own QoL up to now have played only a marginal role in the development of SQoL instruments. The SQoL-instruments referred to above (the Lehman Quality of Life Interview and its adaptions) have not been based on the views of psychiatric patients themselves. One counterexample is the Schizophrenia Quality of Life Scale (SQLS) [20], which is based on – albeit only a few – patient interviews. It has been developed especially for application in clinical studies and is comparable to the SWN-scale (Subjective Well-Being under Neuroleptic Treatment) [21,22]. Comprising only the three subscales *psychosocialmotivation and energysymptoms and side effects*, the SQLS scale’s scope is restricted to effects of antipsychotic drugs, therefore representing only a few fragments of a person’s QoL. It is unlikely to provide a comprehensive picture of the wider range of aspects related to a person’s QoL. A more comprehensive QoL instrument has been developed in France (S-QoL, [23]). Although it has good psychometric properties, its item development was based on only 20 interviews with schizophrenic patients. Therefore, it is unclear whether it is robust enough to represent the vast variation of illness characteristics and settings that characterize the patients’ lives particularly as information on these patients is lacking and it remains unclear to what extent the 20 interviews represent the various views of schizophrenic persons on QoL mentioned above.

Therefore, in our view there is still a need for a QoL-assessment instrument that can reflect the views of persons with schizophrenia based on a broad range of different persons and their respective living conditions. The primary aim of the present comprehensive project was to develop an instrument for the assessment of subjective QoL that is specific to the patients’ perceptions of their own lives. We call this instrument the Quality of Life in Schizophrenia (QLiS) questionnaire. Given our goals, we put substantial effort of our research project into the identification of important life domains specific to the lives of persons suffering from schizophrenia. We included chronically schizophrenic persons in long-term care facilities, chronically schizophrenic persons living in the community as well as schizophrenic patients with a short duration of illness in close proximity to an acute-ward inpatient admission. More than 600 schizophrenic persons contributed to this work, which has been published elsewhere [8,24,25].^a^ We adopted an inductive research strategy in order to better identify those aspects of QoL most important to schizophrenic persons. In this way, we were able to use patients’ own views about their own QoL as a basis on which to develop the QLiS. In this vein, we aimed to capture the patients’ QoL in aggregate terms, i.e. by treating schizophrenic patients as a homogenous group sharing a similar concept of QoL. Still, the QLiS should be close to the themes and aspects of the schizophrenic persons’ life that are of primary importance to them, in a language stemming from their own words.

This article presents an overview of the stages of the development of the QLiS, which comprise different empirical studies, with a focus on the psychometric properties of the QLiS.

## Material and methods

**Figure 1:**
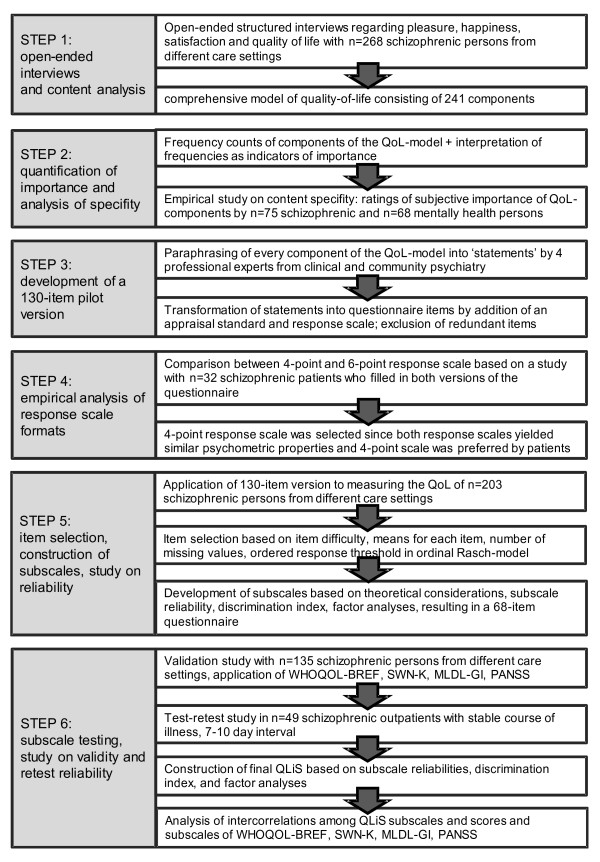
Stages in the development of the Quality of Life in Schizophrenia (QLiS) questionnaire.

Figure 1 provides an overview of the different stages of the development of the QLiS comprising different studies. For the sake of a better readability, these studies will be presented step by step, including their respective methodological approaches. In general, the patients included in the studies had a primary diagnosis of schizophrenia or schizoaffective disorder. All patients suffered from a chronic and/or severe condition, since most of the patients were either living in sheltered/supervised accommodations in the community or they were interviewed during a psychiatric hospital stay. The empirical analysis of the response scale formats (step 4) is the only study in which only schizophrenic hospital patients from a day clinic and hospital wards were included.

With the exception of the application of an ordinal Rasch-model in step 5, the psychometric analyses were all based on the paradigm of classical test-theory (Cronbach’s alpha, item difficulty, scale fit index, test-retest reliability, principal component analysis). All analyses were conducted with SPSS for Windows, except for the ordinal Rasch analyses that were conducted using WINMIRA 32pro [26].

### Developmental steps

#### *STEP 1: Open-ended interviews and content analysis*

##### *Aim*

To identify those aspects of life relevant to QoL from the perspective of schizophrenic persons.

##### *Method*

We conducted structured open-ended interviews with patients from different psychiatric settings (acute hospital ward, community institutional setting, long-term wards). Based on the work of Ludwig [27] we began by asking the patients in personal interviews what had made them happy during the last week, what makes them feel happy in general, what aspects of life they would find very hard to renounce, and what their understanding of QoL was. Subsequently, the subjects were asked to order the aspects they had mentioned with regard to their importance these aspect have on their QoL. Since positive and negative affect have been reported to be (partly) independent in the literature of subjective well-being [28,29], we added questions on what made the person feel unhappy during the last week, what makes him or her unhappy in general, what constituted a bad QoL, and also asked the person to rank these responses according to their importance. On the basis of the answers, we performed a summarizing inductive qualitative content analysis according to Mayring [30]. Respondents’ original statements or paraphrases of their statements were grouped together under provisional headings that were refined to codes. These codes comprise a definition, example statements and, if necessary, inclusion and exclusion criteria for the coding of statements. For example, a code “stigma due to outward appearance” was developed and was used to group statements in which the schizophrenic persons referred to feelings of rejection or stigmatisation because of their outward appearance. Examples of statements that were grouped under this code were “special situations on the streets when people stare at me”, or “when I look ugly because of clothes”. The development of codes took place in an iterative process usually involving at least three members of the research group. The codes were grouped into a hierarchically structured model of QoL, with codes grouped within different QoL-domains.

##### *Results*

We included n = 268 schizophrenic patients, 88 from the acute hospital ward setting, 90 from sheltered community settings, 90 from long-term wards. Fifty-eight percent of the samples were male, mean age was 47 (sd = 17), mean age of onset of illness was 25 (sd = 9) and mean duration of illness was 23 years (sd = 16). Inter-rater reliability of the coding of statements ended up to be 78.4% on code level and 86.3% on domain level (total agreement of three raters). Table 1 presents the QoL domains that serve as subheadings or organizing units of the generated codes. The entire model comprises 241 distinct codes. Further information on the process and results of the content analysis will be detailed in another paper (cf. footnote 1).

**Table 1:** QoL domains and number of codes and coded statements within each domain (in brackets)

**Positive QoL domains**	**Negative QoL domains**
**physical and mental health** (7;142)	**physical and mental illness** (17;364)
including not living in fear, healthy lifestyle, having vitality to live	including suffering from treatment consequences/symptoms/chronic state of disorder, having an addiction
**work/education** (10;165)	**deficits in work life/work functioning** (12;89)
including enjoyable work, work matching ones capabilities, getting money for work	including being unemployed, excessive work demands, conflicts at the work place, bad pay
oral needs* (6;291)	**deprivation of oral needs** (5;46)
including food, coffee, alcohol, smoking	including bad food, not being allowed to smoke
**family** (6;199)	**disturbed family situation** (4;52)
including having contact with ones’ family, parents, own children	including conflicts within family, lack of contact, not being able to care for ones’ family
**social relationships** (16;265)	**tensions in social contacts** (13;216)
including attention and thoughtfulness, friends, positive relations to other patients	including tensions with others, selfish behaviours of others, loneliness, excessive social demands, commiseration with others, social injustice
**partnership/sexuality** (3;112)	**problems with partner/spouse** (3;35)
including contact with spouse, sexual contacts	problems with partner, being separated from partner, having no partner
**spare time activities** (19;481)	**stigma/rejection by environment** (4;47)
comprising a large number of different activities	not being taken seriously, stigma due to outward appearance, being misunderstood
**(social-) psychiatry setting** (10;91)	**suffering related to (social-) psychiatric setting** (8;128)
including aspects of medical treatment, sociotherapy, good care, fewer restrictions due to setting	including feeling imprisoned, restrictions and disturbances due to setting, poor-quality care
**ideas of how to lead a good life** (11;170)	**restrictions on personal way of live** (6;73)
including living like normal people, freedom, routine in life, having future prospects	including worries about future, not being able to cope/inability to live life according to one’s beliefs, restrictions on freedom, dependence
**housing/living environment** (10;117)	**poor housing/living environment** (8;71)
including cleanliness of living environment, safe environment, living independently, having a place to retreat to	including lack of privacy, hectic pace, noise, untidiness/lack of hygiene
**emotional well-being** (9;158)	**lack of emotional well-being** (5;35)
including satisfaction, happiness, love, relaxation, calmness	including unhappiness, bad temper, lack of well-being
**mental lucidity** (4;26)	**difficulties in daily living** (3;46)
learning new things, being creative, societal/political interests	including boredom/monotony, fears/insecurities in daily activities, lack of personal hygiene
**standard of living** (10;182)	**deficient material/social security** (5;87)
including money, financial security, wearing normal clothes, having enough to eat and drink, not being homeless	including poverty, lack of money, being homeless, lack of food, lack of social security
**nature** (3;44)	**aggression/violence** (4;65)
clean natural environment, good weather	physical violence, aggression within psychiatric settings, own aggression
**hygiene and appearance** (2;28)	**deficient self esteem** (2;36)
personal hygiene, a good outward appearance	including poor physical appearance
**faith/religion** (1;32)	**general statements of deficient quality of life** (1;21)

*basic needs related to eating, drinking & smoking.

#### *STEP 2: Quantification of importance and analysis of specifity*

##### *Aim*

To identify those aspects of QoL that are important to schizophrenic persons on the group level and that are specific to their life situations compared to mentally healthy persons.

##### *Method*

Once the whole text material from the qualitative interview from step 1 was assigned to the codes of the QoL-model, we quantified the number of persons who had made a statement on that specific aspect of QoL (and therefore had been grouped under that code). The resulting frequencies of each code served as an indicator of the importance of the respective aspect of QoL for schizophrenic patients on a group level. For the management of the data processing we developed a MS Access database to serve our special needs with regard to coding and counting of the patients’ interview responses [31].

We applied a second approach to identify the relative importance of the respective aspects of QoL. Both schizophrenic patients and mentally healthy persons were asked to rate the *importance* of all components of QoL identified in the content analysis (step 1) on a three-point scale (very important, important, unimportant). A ranked list of importance of aspects of QoL was set up for the schizophrenic and mentally healthy persons, respectively, based on the number of persons in each group rating the respective QoL-aspect as “very important”.

##### *Results*

The frequencies of coded quotations within each QoL domain can be found in Table 1. QoL domains with the highest number of quotations were spare time activities, physical and mental illness, oral needs, social relationships and tensions in social contacts. These domains were followed by family, living standard, ideas how to lead a good life, work/education, physical and mental health, suffering related to (social-)psychiatric setting, and housing/living environment.

In the study using importance ratings, n = 75 schizophrenic persons (community psychiatry patients and acute ward inpatients) as well as n = 67 healthy controls were included. A comparison of the rankings of QoL-aspects for schizophrenic and healthy persons for the first 20 ranks is presented in Table 2.^b^ As the table shows, more mentally healthy subjects rated aspects of QoL as very important compared to schizophrenic persons. We have based the comparison between the importance ratings of schizophrenic and mentally healthy persons on the rank order of aspects of QoL within each group.

**Table 2:** Number of persons rating QoL-aspects rated as “very important” both for schizophrenic and mentally healthy persons, ordered according to schizophrenic persons’ ranking (first 20 items)

	**Schizophrenic persons**		**Mentally health persons**	
	**Rank**	**% rated “very important”**	**Rank**	**% rated “very important”**
health	1	56 (75%)	1	58 (87%)
not being homeless	2	51 (68%)	24	42 (63%)
mental health	3	51 (68%)	4	54 (81%)
physical health	4	48 (64%)	2	57 (85%)
feeling well	5	47 (63%)	15	47 (70%)
having vitality to live	6	47 (63%)	23	43 (64%)
feeling safe/feeling home	7	46 (61%)	10	50 (75%)
being on the right medication	8	46 (61%)	97	12 (18%)
no living in fear	9	46 (61%)	25	40 (60%)
fulfilled, good life	10	45 (60%)	19.5	45 (67%)
harmony within the family	11	45 (60%)	6	52 (78%)
being mentally lucid	12	44 (59%)	11	49 (73%)
experiencing few or no side effects of medication	13	44 (59%)	74	19 (28%)
satisfaction	14	43 (57%)	9	50 (75%)
an environment in which one can feel good	15	42 (56%)	21.5	43 (64%)
having a place to retreat to	16	41 (55%)	26	40 (60%)
love	17	41 (55%)	3	54 (81%)
having enough to eat and to drink	18	40 (53%)	53	27 (40%)
having a housing in which one can feel good	19	40 (53%)	31	37 (55%)
freedom	20	40 (53%)	21.5	43 (64%)

There were some similarities between the groups. Both schizophrenic and healthy persons rated their health as the most important aspect of their QoL. Satisfaction, freedom and mental fitness also received comparably high ranks in both groups. As expected, clear differences were found with regard to those aspects of QoL that are specific to schizophrenia, such as living with fears, getting the right medication, experiencing no or few medication side effects. Schizophrenic patients tended to rank highly those aspects of QoL that dealt with a secure home base, e.g. comfort/cosiness, feeling of being at home, having a place to retreat to, feeling well in ones’ accommodation/home. Some very basic needs were also ranked highly by the schizophrenics, such as having shelter at all, and having enough to drink or to eat. Some examples of aspects of QoL that were of central importance to the healthy persons, but not to a comparable extent to the schizophrenic persons, were (sexual) companionship, family life, outdoor nature, or leading life according to ones’ beliefs. In conclusion, this study provided valuable information as to what aspects of QoL should be selected for a QoL instrument specifically designed for persons suffering from schizophrenia.

#### *STEP 3: Development of a 130-item pilot version*

The components of QoL derived from the content analysis that were found to be relevant to schizophrenic persons were selected. Four professional experts from clinical and community psychiatry were asked to convert these components into single statements. The experts were provided with the label of the respective category, a definition of the category, and all original quotations coded into this category. Their task was to express the content of the category in a single statement, while being as faithful to the content, language and tone of the original responses as possible. In accordance with the principles of a delphi-approach, the experts wrote their statements completely independently from one another so as to yield at least four different statements within each category.

The resulting statements were reviewed by our research team and the most appropriate statements were selected. During this stage, each statement was transformed into a questionnaire item by adding an appraisal standard and a response scale. For some statements, a reference to the person him-/herself had to be added (“I feel that…”, “I suffer from…”, “…makes me…”, “I have difficulties in…”). At this point, we paid a lot of attention to developing simple, easy to understand, unambiguous items and asked experts from the field of questionnaire development to comment on them. We made our final item selection on the basis of the results of step 2 (see above), redundancy of items and considering the number of items per domain in order to avoid inappropriate weighting of specific facets of QoL. This process resulted in a 130-item pilot version of the questionnaire.

#### *STEP 4: Empirical analysis of response-scale formats*

Background and aim: There is no simple procedure for deciding on the number of response categories for a QoL questionnaire for schizophrenic patients. While questionnaires such as the Nottingham Health Profile apply dichotomous response formats, the widely used Lancashire Quality of Life Profile and the Manchester Short Assessment of Quality of Life (MANSA) employ seven response categories. Important criteria for the selection of a response format include the psychological meaningfulness of the categories, feasibility for cognitively disturbed patients, avoidance of response sets, enough possibilities for patients to distinguish between different QoL experiences, and, of course, patients’ preferences.

While a higher number of response categories should result in a higher amount of variance in the data, it is not clear whether this variance is due to a better representation of the measured construct (valid variance) or mere method variance. In one study, Chang [32] was able to show that a 6-point Likert scale was not superior to a 4-point Likert scale in the prediction of an external validity criterion. Also, we had to decide whether to use an uneven or an even number of response categories, the latter avoiding a mid-point category. A mid-point category might serve as a neutral response for those respondents who are undecided on a given point, but it may also increase the likelihood that they choose this mid-point category. We decided to omit the mid-point category so as to yield a “forced choice” situation, in which the person either has to agree or disagree on item content.

Because we excluded the possibility of a 2-point response format as too restrictive, we had to choose between a 4-point and a 6-point response scale. We could not, however, base our decision on theoretical grounds. Therefore we conducted an additional empirical study with the aim of comparing the 4-point and 6-point response scales with regard to psychometric characteristics and preferences by schizophrenic patients.

##### *Methods*

Each scale point of the 4-point and 6-point response scales was labeled by a verbal description. For example, the scale points of the 4-point response scale were labeled “disagree”, “rather disagree”, “rather agree”, “agree”. Schizophrenic patients from the outpatient clinic or the psychiatric hospital ward completed a questionnaire in which a selection of 52 items had to be rated both on a 4-point and 6-point scale (arranged in two blocks that were presented in chance order so as to rule out order effects). Afterwards the patients were asked which version they preferred and to give reasons for their decision. Also, we asked the patients to give additional feedback on the questionnaire (comprehensibility, item content, layout etc.), which then further informed modifications of the pilot version.

##### *Results*

n = 32 schizophrenic patients participated in this study. The mean age of the participants was 40.7 (sd = 12.9), 59% were female. The empirical distribution characteristics of the two response scales were very similar. The empirical means for both scales (2.80 vs. 3.96) were higher than the respective theoretical means (2.5 vs. 3.5). The deviance of the empirical mean from the theoretical mean divided by the number of categories was nearly identical. Fourteen patients (44%) favoured the 4-point response format, eight (25%) the 6-point response format, five patients (16%) did not prefer either, and an additional five patients did not respond to this question. While the patients favouring the 6-point response format emphasized the enhanced possibilities to give a more detailed answer to the questions, all of the patients favouring the 4-point scale referred to its better fit with their cognitive abilities (“concentration”, “more comprehensible”, “makes it easier to decide”).

##### *Discussion*

The empirical characteristics of the response distributions did not favour one response format over the other and the patients’ preference tended toward the 4-point response format. In addition, answering a complete questionnaire with a lot of items demands great efforts for schizophrenic persons who often have cognitive limitations (e.g. problems with attention). Therefore, they should benefit from a simpler 4-point-scale. Hence, we decided to include the 4-point response scale in the 130-items pilot version of the questionnaire. Numbers from 1 to 4 were added to the 4-point response scale at this point, with a higher number representing a higher level of QoL. Also, different visual clues have been integrated into the design of the questionnaire so as to make it easier for the patients to fill it in.

#### *STEP 5: Item selection, construction of subscales, study on reliability*

##### *Aim*

To construct subscales with good psychometric properties.

##### *Method*

Application of the 130-item pilot version to the measurement of the QoL of schizophrenic persons from different psychiatric settings. We used two versions of the pilot instrument with different item orderings so as to control for order effects. To identify possible order effects we compared item means and scale means between the two versions. All items with item difficulties > .85 or < .15 were to be excluded from further analysis, because these items do not provide sufficient information on a group level. Items were also analysed by means of an ordinal Rasch model in order to identify violations of threshold order of the response scale. Since the general aim of the project was to develop an instrument that is able to represent the QoL specific to schizophrenic patients, it was not our intention to yield one single index but instead a profile of important facets of a schizophrenic person’s life. Therefore we aimed to develop a number of theoretically and empirically sound subscales. The development of the subscales was a recursive process based on theoretical considerations, results of reliability analysis (internal consistency), factor analyses, item discrimination indices, and analyses of floor- and ceiling effects. We also performed an analysis of response sets. We analysed the data with regard to “yea-saying” (acquiescence, answering affirmatively independent of content), a tendency to rate at the extremes (i.e. only using the outer categories *agree* or *disagree*), a tendency to use only the inner categories (*rather agree* and *rather disagree*). If a respondent filled in more than 90% of the items in accordance to the definition of a respective response set, this person was identified as applying a response set.

##### *Results*

n = 203 schizophrenic patients from psychiatric hospitals (67%) and community psychiatric institutions (31.5%; 1.5% provided no information) from the central and northern parts of the German state of Hesse were surveyed. Sociodemographic and clinical characteristics of the sample are presented in Table 3.

**Table 3:** Characteristics of the samples of schizophrenic persons (step 5 and step 6)

	**Sample step 5subscale construction and reliabilityn = 203**	**Sample step 6 subscale testing and validityn = 135**
Age (in years)	37.3 (±10.2)	37.4 (±9.8)
Sex (% female)	50%	46%
Married/partnership	25%	13%
Education (highest completed)		
none	3%	4%
special education	3%	2%
intermediate	30%	25%
technical high school	34%	41%
university entrance qualification	27%	27%
other	2%	
Normal or sheltered work	26%	29%
Age at onset of illness	23.5 (±9.5)	25.6 (±8.2)
Number of psychiatric admissions	5.5 (±5.1)	7.1 (±7.6)
Cumulative duration of hospitalisation (months)	30.7 (±45.0)	25.2 (±31.4)
Global Assessment of Functioning Scale (GAF)	n.a.	51.7 (±18.0)
Psychopathology (PANSS)	n.a.	
positive symptoms		13.2 (±5.5)
negative symptoms		15.0 (±5.5)
general psychopathology		28.7 (±7.3)
total score		57.0 (±15.1)

*PANSS* Positive and Negative Syndrome Scale.

Analysis of item difficulties identified 15 items, which all had item difficulties < .15, i.e. they were very easy to agree with. The remaining items were checked for extreme mean values and number of missing values. Three additional items were excluded due to unordered threshold parameters in ordinal Rasch-modelling of preliminary subscales.

One person (0.5%) was found to have applied a “yea-saying” (acquiescence) response set (complying with items independent of content), six persons (3%) had a tendency to rate at the extremes, and 28 persons (14%) showed a tendency to rate using only the inner categories. However, excluding these patients from analysis of subscales did not alter the psychometric properties of the pilot instrument.

The development of the subscales resulted in 16 distinct subscales containing 68 items. The subscales were found to have satisfactory to good psychometric properties (Table 4). No difference could be found between the two versions with different item ordering with regard to item and subscale means.

**Table 4:** Psychometric characteristics of the QLiS subscale (results from study in step one; n = 203), validation study in step 6 (n = 135) and retest-study in step 6 (n = 49)

**QLiS-subscales*example of item***	**# items**	**mean (std.-dev.)**	**scale-fit (%)**	**floor/ceiling effects (%)**	**internal consis-tency (α)**	**test-retest-reliability**
social contacts *There are too few people visiting me.*	5	4.42 (±2.22) 4.47 (±2.24)	98.2 96.4	3.4/1.5 4.4/1.5	.71.63	n.a..73
appreciation by others *I feel respected by other people.*	4	6.47 (±2.36) 6.37 (±2.13)	100 97.7	2.5/9.9 1.5/5.9	.78 .70	n.a. .72
relationship to family *My family provides me with backing and strength.*	3	6.15 (±2.96) 5.90 (±2.55)	100 100	6.9/17.2 3.0/8.1	.82 .71	n.a. .82
appraisal of pharmacotherapy *I feel no interests due to my medication.*	6	5.60 (±2.25) 5.97 (±2.11)	100 100	2.0/3.9 0.7/2.2	.77 .71	n.a. .79
appraisal of psychopathological symptoms *I suffer from inner unrest.*	6	5.16 (±2.53) 5.58 (±2.40)	100 96.7	2.5/3.4 0.7/2.2	.84 .82	n.a. .76
cognitive functioning *I feel alert and can think straight.*	5	5.28 (±2.37) 5.49 (±2.31)	100 98.5	2.0/3.9 0.7/1.5	.81 .79	n.a. .83
abilities to manage daily living *I feel dependent on others.*	4	6.23 (±2.38) 6.42 (±2.43)	100 100	1.0/10.3 0.7/14.1	.75 .74	n.a. .82
appraisal of accommodation/housing *I have sufficient opportunities to retreat to.*	5	6.04 (±2.23) 6.14 (±2.17)	100 100	1.5/5.9 0.7/3.7	.73 .73	n.a. .77
financial situation *I do not have enough money for ordinary things, such as decent clothes, cigarettes.*	4	5.17 (±2.59) 4.72 (±2.65)	100 100	3.4/4.4 5.2/3.7	.75 .77	n.a. .87
leading a “normal“ life *I am leading a “normal” life like everyone else is.*	3	4.52 (±2.58) 4.56 (±2.60)	97.7 96.7	6.9/5.4 3.7/3.7	.70 .70	n.a. .81
confidence *I have a great deal of self-confidence.*	4	5.73 (±2.42) 5.74 (±2.28)	100 100	2.0/6.9 1.5/3.7	.75 .74	n.a. .76
global life-satisfaction *I lead a happy life.*	3	4.26 (±2.56) 4.64 (±2.75)	100 100	10.0/3.4 8.9/4.4	.80 .88	n.a. .82

subscale means from 0 (worst possible QoL) - to 10 (best possible QoL), theoretical mean = 5 scale-fit: based on the analysis of every item-times-subscale correlation; it is defined as the number of corrected item-subscale correlations that are higher in absolute value compared to the correlations of the subscale items with other subscales in relation to all possible item-subscale-correlations floor/ceiling effects: number of subjects with the lowest/highest possible subscale value internal consistency: Cronbach’s alpha test-retest-reliability: Pearson correlation coefficient between two assessments in stable non-hospitalised patients 7–10 days apart.

#### *STEP 6: Subscale testing, study on validity and retest reliability*

##### *Aim*

1. To analyse the psychometric characteristics of the subscales of the questionnaire developed in step 5 and to modify the subscales if necessary. 2. To analyse the convergent and discriminative validity of the instrument.

##### *Methods*

A cross-sectional survey applying a 68-item version in n = 135 schizophrenic persons from different psychiatric settings was conducted. For the resulting subscales we calculated the mean and standard deviations, scale fit (the number of corrected item-subscale correlations that are higher in absolute value compared to the correlations of the subscale items with other subscales in relation to all possible item-subscale-correlations), occurrence of floor and ceiling effects (number of subjects with the lowest/highest possible subscale value), and reliability in terms of internal consistency (Cronbach’s Alpha).

In order to analyse different aspects of the validity of the QLiS, other QoL instruments (WHOQOL-bref [17,33], short version of the SWN [21,22], satisfaction items of the German version of the LQLP (13,14)) were applied on the same subjects and at the same time point as the QLiS, so as to yield correlational evidence on how specific or redundant the information provided by the QLiS is. In addition, patients’ psychopathology was assessed by means of the Positive and Negative Syndrome Scale (PANSS) [34], and indicators representing the objective living situation of the subjects were also included.

We transformed the original subscale scores ranging from 1 to 4 with a midpoint of 2.5 to a score range from 0 to 10, with a midpoint of 5 by the following formula:

(1) $$newscale=\frac{\left(x-1\right)}{3}\times 10$$

(x = old scale mean ranging from 1 to 4)

This transformation was made to facilitate easier interpretation of the subscale scores.

In addition to the cross-sectional survey we conducted a retest-reliability study with schizophrenic persons, including clinically stable outpatients. We aimed for an interval of one to two weeks between assessments. We decided on this interval in order to minimize the risk of any major changes in the lives of the patients interfering with the results and also to minimize recall effects that might be present in shorter intervals. Retest-reliability was computed by means of the Pearson-correlation coefficient of the subscale scores between both time points.

##### *Results*

Sociodemographic and clinical characteristics of the sample are presented in Table 3.

Modification of subscales: six subscales were left unchanged following step 6, *social contacts*, *appreciation by others*, *relationship to family*, *appraisal of accommodation/housing*, *financial situation* and *leading a “normal” life*. Only slight modifications were made to the subscales regarding *cognitive functioning*, *confidence* and *general life satisfaction*. Items from the subscales formerly called “side-effects of medication” and “physical well-being” were merged to the subscale *appraisal of pharmacotherapy*. In the same vein, items from the former subscales “mental health” and “depression” were merged to the subscale *appraisal of psychopathological symptoms*. The subscale *ability to manage daily living* was based on the former subscale “independent living”. From a theoretical perspective, this last subscale is of high importance for a QoL assessment in schizophrenic persons. This subscale had strong empirical associations with *cognitive functioning* and *appraisal of psychopathological symptoms*. For the QoL domain ‘work’ no psychometrically sound subscale could be developed, possibly due to the heterogeneity of the persons’ work situations. This will be an important task for further development of the QLiS. For the time being, two work-related items were added to the QLiS, one for people who have a job, one for those who are unemployed.

Table 4 comprises the psychometric characteristics of the twelve subscales based on two studies from step 5 and step 6. In sum, the QLiS comprises 52 items (plus two items in the work domain, see above), the number of items per subscale ranges from 3 to 6. The mean values of the subscales cluster around the theoretical mean of 5, with a tendency to the positive side of the scale (3 subscales negative vs. 8 subscales positive in both studies). The scale fit index represents the degree to which the items of a subscale are a useful part of the respective subscale (see Table 3). For 7 of the 12 subscales we found a 100% fit in both studies. Two scales have shown a lower level of fit in both studies (*social contacts* and *leading a “normal” life*), although they still had a high absolute value (minimum scale fit score = 96.4%). Floor and ceiling effects (defined as the proportion of persons with the lowest or highest possible score in relation to all persons) were more prominent in the subscales with fewer items. The most excessive value was found in the family subscale (ceiling effect 17.2%), although this value dropped to below 10% in the validity study. The subscale *abilities to manage daily living* was found to have higher ceiling effect values, too. Floor effects seemed to be negligible, the highest values being found in the *general life-satisfaction* subscale (10.0% and 8.9%). The internal consistency as an important indicator of subscale reliability was found to be greater or equal to Cronbach’s α = .70 with the exception of one subscale.

Results of the test-retest-reliability study are also found in Table 4. The time-points of the assessments were 7–10 days apart. All of the reliability coefficients were above r_tt_ = .70.

The assignment of items to subscales means that some important aspects of a schizophrenic person’s QoL do not represent a whole subscale but can be found under another heading. Spare time activities have become part of the *accommodation/housing* subscale representing sufficient opportunities for spare time activities. The aspect of QoL concerning religion/beliefs has been integrated in the *confidence* subscale. The aspect of QoL concerning security has been integrated into the *financial situation* subscale. Aspects of physical health were dominated by the items appraising the pharmacological treatment, and therefore items of physical health did not constitute a distinguishable subscale.

The results on the convergent and discriminate validity of the QLiS have been presented in a separate paper [35].

## Discussion

The key feature of the development of the QLiS is the step-by-step, inductive procedure applied, which was founded on a large number of schizophrenic patients’ views on their own QoL. Moreover, it incorporated different means to preserve the specifity of their views. In terms of rigor and comprehensiveness, we know of no comparable approach in the field of subjective QoL research in psychiatry.

The face validity and specifity of the QLiS can be demonstrated by the subscales themselves that have emerged during the developmental process. The subscale *appreciation by others* comprises experiences of stigma and being respected by others as a fellow citizen. This important aspect to the schizophrenic persons’ life has not been explicitly conceptualised in other QoL instruments. However, in his review of determinants of QoL in schizophrenia Hanson, too, has argued for an important role of stigma in QoL [36], and it has also emerged as a central theme in another – albeit small – qualitative study [37]. Perceived social support has been reported to be positively associated with general SQoL [38].

Especially with regard to the pronounced relationship between depression and SQoL, there has been a strong argument against confounding clinical symptoms and QoL (e.g. [36]). However, some prominent symptoms of schizophrenia have a considerable impact on the *persons’ sense* of well-being. This is represented by a subscale where patients appraise the impact the symptoms on their QoL (*appraisal of psychopathological symptoms*). The possible impact of pharmacologic treatment on the persons’ life is reflected in the subscale *appraisal of pharmacotherapy*. Both themes have also emerged in the qualitative study by Gee et al. [37]. Another more general aspect of QoL cannot be found in other QoL instrument: *leading a normal life*. There had been some controversy in our research group as to how to incorporate this aspect into the QLiS. The comparison of ones’ own life to a normal life implies a general tendency to change ones’ frames of references and expectations in the course of a chronic mental illness. However, different codes that were developed in the qualitative phase of this project express the notion of *leading a “normal” life* (see also [37]) that the inclusion of this aspect in the QLiS was deemed essential.

Common approaches to QoL-assessment in the mentally healthy population are based on the assessment of QoL in different life domains. These domains have also emerged in our approach to a large degree. The appraisal of *social contacts*, of the *relationship with the family*, of the *financial situation* or of one’s *accommodation/housing* have to be regarded as such life domains. However, as could be shown by examples of the items that constitute these subscales, these subscales represent aspects of life that appear to be very specific to the schizophrenic persons’ lives.

The subscale *cognitive functioning* can be regarded as similar to related subscales found in other instruments, e.g. the mental-functioning subscale of the SWN [21,22]. The subscale *general life satisfaction* was set up to represent the evaluation of a person’s life from a very general perspective. This is in accordance with the construct-validation approach of Pukrop and colleagues, who explicated a central facet of QoL called *general QoL*[39]. This facet can also be found in the Quality of Life Interview, the Lancashire Quality of Life Profile and the WHOQOL instrument. This subscale of the QLiS comprises aspects of satisfaction, happiness and general feelings of well-being.

There are QoL domains of other QoL instruments that did not emerged as a subscale of the QLiS, including spare-time activities, religion/beliefs, safety and aspects of physical health, as reported in the results section of step 6. Last but not least, the work/education domain is still in need of further development, since, although it is of major QoL importance to a lot of patients, it did not emerge in a psychometrically sound subscale possibly due to the heterogeneity of the persons’ work situations. This aspect of the QLiS therefore needs further development.

Based on criteria of classical test theory, from a psychometric perspective the QLiS is a well-founded, objective and reliable instrument with a high level of content validity. In line with other QoL research in severely mentally ill patients, low QoL ratings were found in the domains of social contacts, finances and general life-satisfaction, as well as for the subscale *leading a “normal” life*. This stands in contrast to the findings of generic QoL scales that repeatedly found high satisfaction in schizophrenic patients – even under poor living conditions [7,8,40]. It should be noted, that despite a tendency to rate ones’ own live rather positively, most subscales’ means of the QLiS centre around the theoretical mean of five.

The development of the QLiS contributes to the discussion of the validity of the SQoL construct in schizophrenic persons. We now have a firm basis to claim that we can assess the most important aspect of schizophrenic persons’ QoL. The QLiS is not a generic scale and therefore not suitable for comparisons between different patient groups beyond schizophrenic persons. Nonetheless, it should be highly suitable in the assessment and appraisal of the profiles of SQoL from the perspective of the schizophrenic persons on a group level in psychiatric research. The QLiS has yet to demonstrate its usefulness in future application. The original version of the QLiS is in the German language; a preliminary English translation exists but still has to be validated. The QLiS can be obtained from the authors upon request.

## Conclusions

Based on a thorough development process, a questionnaire on subjective quality of life in schizophrenia, the QLiS, has been made available. It is characterized by a high level of content validity and a sound psychometric basis.

## Endnotes

^a^An English language publication is presently under review (Meyer T, Gallhofer B, Franz M: What do schizophrenic persons mean by ‘quality of life’? A content-analysis of 268 open-ended interviews.)

^b^An extensive documentation of the results of this study has been done in a psychological thesis by Dimmerling C (2000) Spezifität subjektiver Vorstellungen schizophren erkrankter Menschen zur Lebensqualität. Ein Vergleich schizophrener Patienten mit Gesunden (Specifity of subjective theories of schizophrenic persons about quality of life. A comparison between schizophrenic patients and healthy persons). Unpublished thesis for the attainment of the diploma in psychology at the psychological department of the Justus-Liebig University Giessen, Germany.

## Competing interests

The authors declare that they have no competing interests.

## Authors’ contributions

MF has initiated, planned and supervised the whole process of the QLiS development, was involved in all phases of the conduct of the study and critically revised the draft of the article. MF was responsible for conducting the studies from step 3 to step 6 and the statistical analyses in these steps and has critically revised the draft of the article. BG made substantial contributions to the conception and design of the whole study and he has been involved in revising the manuscript critically. TM was involved in the planning of stages 2 to 6 and in conducting all stages of the QLiS development including the statistical analyses. He also wrote the first draft of the paper. All authors read and approved the final manuscript.
